# Genome-Wide Identification and Expression Analysis of the *CsCAMTA* Gene Family in Tieguanyin Tea Plants Under Heat Stress

**DOI:** 10.3390/cimb48060597

**Published:** 2026-06-05

**Authors:** Zijia Cui, Hua Wu, Zhicheng Yang, Bohao Xu, Fan Jiang, Rien Lai, Lu Han, Ciding Lu, Dandan Li, Kehui Zheng

**Affiliations:** 1 School of Modern Forestry, Jiangxi Environmental Engineering Vocational College, Ganzhou 341000, China; zeocui@126.com (Z.C.); xu1727276519@163.com (B.X.); 19579960069@163.com (F.J.); 17719100918@163.com (R.L.); 18687184962@163.com (L.H.); wayphx@163.com (C.L.); 2 College of Food Science, Fujian Agriculture and Forestry University, Fuzhou 350002, China; w2918083657@163.com; 3 College of Computer and Information Sciences, Fujian Agriculture and Forestry University, Fuzhou 350002, China; 18359797810@163.com

**Keywords:** Tieguanyin tea plant, *CAMTA* transcription factor, heat stress, gene expression analysis, stress resistance mechanism

## Abstract

Tieguanyin (*Camellia sinensis* cv. Tieguanyin) is an important oolong tea cultivar in China, and heat stress has become a major environmental constraint affecting its growth and productivity. Calmodulin-binding transcription activators (*CAMTAs*) are important transcription factors involved in calcium/calmodulin-mediated signaling and plant responses to environmental stresses. However, systematic knowledge of the *CAMTA* gene family in Tieguanyin remains limited. In this study, 20 *CsCAMTA* genes were identified from the Tieguanyin genome and characterized based on their physicochemical properties, phylogenetic relationships, conserved motifs, gene structures, chromosomal distribution, collinearity, promoter cis-acting elements, and functional annotation. The 20 *CsCAMTA* genes were unevenly distributed across eight chromosomes, and collinearity analysis suggested that segmental duplication may have contributed to the expansion of this gene family. Conserved motif and domain analyses indicated that *CsCAMTA* proteins retained typical structural features of *CAMTA* transcription factors, including CG-1, ANKYR, TIG, and *CaMBD*/IQ-related regions. Promoter analysis showed that *CsCAMTA* genes harbored multiple cis-acting elements related to hormone responsiveness, stress response, light response, and growth regulation. Furthermore, qRT-PCR analysis of 18 representative *CsCAMTA* genes under 40 °C heat treatment revealed distinct temporal expression patterns, suggesting that different *CsCAMTA* members may respond to heat stress at different stages. Several genes, such as *CsCAMTA*2, *CsCAMTA*10, and *CsCAMTA*16, showed marked transcriptional changes and may represent candidate heat-responsive genes in Tieguanyin. These results provide a systematic overview of the *CsCAMTA* gene family and lay a foundation for further functional studies of heat stress responses in Tieguanyin.

## 1. Introduction

Tieguanyin (*Camellia sinensis* cv. Tieguanyin) is a representative oolong tea cultivar in China. Owing to its distinctive aroma profile and favorable processing characteristics, Tieguanyin has become an important resource for the high-value development of the tea industry [1,2,3,4]. However, in recent years, the intensification of global climate change has increased the frequency of extreme weather events, including drought, high temperature, and strong radiation, which seriously threaten the yield and quality stability of Tieguanyin in its major production areas [5]. Abiotic stresses can induce the overproduction of reactive oxygen species (ROS), impair the photosynthetic system, and disrupt secondary metabolic networks, thereby altering the balance of tea polyphenols and amino acids and ultimately affecting tea flavor quality [6]. Therefore, elucidating the molecular regulatory networks underlying stress responses in Tieguanyin, especially the core components of stress signal transduction, is important for variety improvement and the sustainable development of the tea industry.

Calcium signaling is a central regulatory system in plant responses to environmental stresses. As key components of this system, calmodulin-binding transcription activators (*CAMTAs*) play important roles in regulating plant environmental adaptation [7,8,9]. *CAMTA* proteins typically contain conserved CG-1 DNA-binding domains, ANK repeats, and IQ motifs [10]. They can recognize the cis-acting element (A/C) CGCG box and participate in stress responses by regulating downstream gene expression networks associated with abscisic acid (*ABA*), salicylic acid (*SA*), and heat shock proteins (*HSPs*) [11]. In model plants, *AtCAMTA*3 enhances cold tolerance in Arabidopsis by activating the *CBF/DREB1* cold-response module [12,13,14]. In soybean, *GmCAMTA12* regulates stomatal movement through an ABA-dependent pathway and improves drought resistance [15,16]. *GhCAMTA*11 in cotton has been identified as a key regulatory factor involved in heat stress responses, and its overexpression significantly enhances plant heat tolerance [17]. In addition, *SlCAMTA*4 in tomato affects plant resistance to pathogens by regulating the jasmonic acid (JA) signaling pathway, while *OsCAMTA*1 in rice is involved in salt stress responses [18,19]. These studies indicate the evolutionary conservation and functional diversity of the *CAMTA* family in plant stress signaling networks.

In recent years, with the development of tea plant genomics and transcriptomics, several *CsCAMTA* members have been identified in tea plants, and their expression patterns have shown tissue-specific and treatment-dependent characteristics under low-temperature, drought, and *ABA* treatments [10]. For example, the expression levels of *CsCAMTA*3 and *CsCAMTA*5 in the cold-tolerant cultivar “Longjing 43” were higher than those in the cold-sensitive cultivar “Da Bai Mian”, suggesting their potential involvement in tea plant cold adaptation. Furthermore, studies on woody plants have expanded our understanding of *CAMTA* function. For example, in Phoebe bournei, 17 *PbCAMTA* genes form a dynamic co-expression network under drought, high-temperature, and salt stresses, with *PbCAMTA*2, *PbCAMTA*12, and *PbCAMTA*16 identified as core stress-response nodes [9]. In Populus trichocarpa, *PtCAMTA*7 enhances drought tolerance by regulating antioxidant enzyme activity and increasing ROS-scavenging capacity [20]. These findings provide useful references for understanding stress adaptation mechanisms in woody plants, including tea plants.

Although *CAMTA* genes have been reported in several plant species, systematic identification and heat-responsive expression analysis of the *CAMTA* gene family in Tieguanyin tea plants remain limited. In this study, we performed genome-wide identification of *CsCAMTA* genes in Tieguanyin and analyzed their phylogenetic relationships, physicochemical properties, conserved motifs, gene structures, chromosomal distribution, duplication events, promoter cis-acting elements, and functional annotations. In addition, 18 representative *CsCAMTA* genes were selected for qRT-PCR analysis under heat stress. This study aimed to clarify the basic characteristics of the *CsCAMTA* gene family in Tieguanyin and to identify potential candidate genes involved in heat stress responses.

## 2. Materials and Methods

### 2.1. Data Sources

The genome assembly and genome annotation files of Tieguanyin tea plant were retrieved from the Genome Warehouse of the China National GeneBank Database under accession number GWHASIV00000000 [21]. To support comparative analyses, genome sequences and corresponding annotation files of *Arabidopsis thaliana*, *Triticum aestivum*, *Zea mays*, *Nicotiana tabacum*, *Glycine max* (L.) Merr. and *Solanum lycopersicum* were downloaded from public genome databases, including TAIR (https://www.arabidopsis.org/, accessed on 3 May 2025) and Ensembl Plants (https://plants.ensembl.org/index.html, accessed on 3 May 2025). The reported CAMTA protein sequences of A. thaliana were collected from PlantTFDB (https://planttfdb.gao-lab.org/, accessed on 25 May 2025) and used as query sequences for subsequent identification. The hidden Markov model profile of the CAMTA-associated conserved domain was downloaded from the Pfam database (http://pfam.xfam.org/, accessed on 3 May 2025) and used for domain-based screening of candidate CAMTA members.

### 2.2. Identification and Physicochemical Properties of CAMTA Gene Family

To identify *CAMTA* family members in Tieguanyin, two complementary strategies were used. First, the known A. thaliana *CAMTA* protein sequences were used as queries to search against the Tieguanyin protein database using the BLAST module in TBtools (version 2.310). Second, the HMM profile of the *CAMTA* conserved domain was employed to perform an HMMER-based search against the Tieguanyin proteome. Candidate proteins identified by both approaches were merged and redundant sequences were removed. The conserved domains of the remaining candidates were further examined using the NCBI Conserved Domain Database to confirm the presence and completeness of characteristic *CAMTA* domains. Sequences lacking essential conserved regions or showing obvious annotation abnormalities were excluded from further analysis. Multiple sequence alignment of confirmed *CsCAMTA* proteins was performed using DNAMAN (version 9.0) to examine sequence conservation among family members [22]. The amino acid length, molecular weight, theoretical isoelectric point, instability index, and other physicochemical parameters of *CsCAMTA* proteins were predicted using the ExPASy ProtParam (https://web.expasy.org/protparam/, accessed on 3 May 2025) [23], and the resulting data were summarized using WPS Excel (version 12.1.0).

### 2.3. Analysis of Gene Structure and Conserved Motifs

The gene structure information of *CsCAMTA* genes was extracted from the genome annotation files in GFF3/GTF format. The exon–intron organization of each *CsCAMTA* gene was visualized using the Gene Structure View module in TBtools. To identify conserved motifs in *CsCAMTA* proteins, the full-length amino acid sequences of *CsCAMTA* family members were submitted to the MEME online program (https://meme-suite.org/meme/, accessed on 6 May 2025), with the maximum number of motifs set to 10. The distribution of conserved motifs was further visualized using TBtools. Conserved protein domains were analyzed using the NCBI Conserved Domain Database (CDD) (https://www.be-md.ncbi.nlm.nih.gov/cdd/, accessed on 6 May 2025), and the domain organization of *CsCAMTA* proteins was displayed using the Visualize NCBI CDD Domain Pattern function in TBtools. The phylogenetic tree, conserved motifs, conserved domains, and gene structures were integrated to compare the structural conservation and divergence among *CsCAMTA* family members.

### 2.4. Chromosome Mapping and Collinearity Analysis

The chromosomal locations of *CsCAMTA* genes were obtained from the Tieguanyin genome annotation file and plotted using the Gene Location Visualize function in TBtools. The physical positions of *CsCAMTA* genes on chromosomes were adjusted according to chromosome length and gene coordinate information. Gene duplication events within the Tieguanyin genome were analyzed using MCScanX of TBtools, and duplicated *CsCAMTA* gene pairs were visualized using the Circos function in TBtools [24]. To further explore the evolutionary relationships of *CAMTA* genes across species, interspecific synteny analyses were performed between Tieguanyin and *A. thaliana*, *T. aestivum*, *Z. mays*, and *N. tabacum* using MCScanX. The collinear gene pairs were then visualized to evaluate the conservation and divergence of *CAMTA* genes among different plant species.

### 2.5. Phylogenetic Tree Construction

The full-length *CAMTA* protein sequences from Tieguanyin, soybean, tobacco, tomato, and *Arabidopsis thaliana* were used for phylogenetic analysis. Multiple sequence alignment was first conducted, and the resulting alignment file was used to construct a phylogenetic tree with the One Step Build a NJ Tree module in MEGA12. Bootstrap analysis was performed with 1000 replicates to evaluate the reliability of each branch. The final phylogenetic tree was exported and further refined using Adobe Illustrator CC 2018 [25]. Based on the clustering relationship between *CsCAMTA* and *AtCAMTA* proteins, *CsCAMTA* members were classified into different subgroups.

### 2.6. Promoter Cis-Acting Element Analysis

For promoter analysis, the 2000 bp upstream sequences of *CsCAMTA* genes were extracted from the Tieguanyin genome using the Sequence Extract function in TBtools. These upstream sequences were submitted to PlantCARE to predict putative cis-acting regulatory elements. The identified cis-elements were classified according to their functional annotations, including elements related to hormone responsiveness, abiotic stress response, light response, and growth regulation. The distribution of major cis-elements in *CsCAMTA* promoter regions was visualized using the Simple BioSequence Viewer module in TBtools.

### 2.7. Tissue-Specific Expression Analysis

To investigate the expression characteristics of *CsCAMTA* genes in different tissues, transcriptome-derived FPKM values of roots, stems, and leaves of Tieguanyin were collected and analyzed. The expression matrix of *CsCAMTA* genes was organized and normalized before visualization. Expression heat maps were generated using the HeatMap module in TBtools, and the final figures were adjusted for clarity. Differences in expression levels among roots, stems, and leaves were used to infer the potential tissue-specific functions of *CsCAMTA* genes.

### 2.8. Prediction of Secondary and Tertiary Protein Structures

The secondary structures of *CsCAMTA* proteins were predicted using SOPMA (https://npsa.lyon.inserm.fr/cgi-bin/npsa_automat.pl?page=/NPSA/npsa_sopma.html, accessed on 20 December 2025), including the proportions of α-helix, extended strand, β-turn, and random coil. For tertiary structure prediction, the amino acid sequences of *CsCAMTA* proteins were submitted to SWISS-MODEL for homology modeling. The predicted three-dimensional structures were compared among different *CsCAMTA* subgroups to evaluate potential structural differences and functional divergence within the family.

### 2.9. Plant Materials and Heat Treatment

One-year-old Tieguanyin tea seedlings with uniform growth status were used as experimental materials. The seedlings were obtained from Xiping Town, Anxi County, Fujian Province, China, and maintained under controlled conditions in an artificial climate chamber before stress treatment. For heat treatment, healthy and consistently growing seedlings were transferred to a growth chamber set at 40 °C. Leaf samples were collected at 0, 4, 8, 12, and 24 h after treatment, with the 0 h samples serving as the control. For each time point, three independent biological replicates were prepared, and each biological replicate consisted of pooled leaves collected from at least three individual seedlings. All samples were immediately frozen in liquid nitrogen after collection and stored at −80 °C until RNA extraction.

### 2.10. RNA Extraction, cDNA Synthesis and qRT-PCR Analysis

Total RNA was extracted from heat-treated Tieguanyin tea leaf samples using an RNA extraction kit (RA106-01, Beijing Biomarker Technologies Co., Ltd., Beijing, China) according to the manufacturer’s instructions. The concentration and purity of RNA were assessed using a NanoDrop spectrophotometer by Thermo Fisher Scientific (Waltham, MA, USA), and RNA integrity was checked by agarose gel electrophoresis. High-quality RNA was then used for first-strand cDNA synthesis using the MT403-01 reverse transcription kit (Beijing Biomarker Technologies Co., Ltd., Beijing, China) following the manufacturer’s protocol.

To examine the transcriptional responses of *CsCAMTA* genes under heat stress, 18 representative *CsCAMTA* genes were selected for qRT-PCR analysis. Gene-specific primers were designed in the non-conserved regions of the target genes using TBtools software, and the primers were synthesized by Fuzhou Boshan Biotechnology Co., Ltd. (Fuzhou, China).

qRT-PCR was performed using SYBR Green qPCR Master Mix by Thermo Fisher Scientific (Waltham, MA, USA) on a real-time PCR detection system. The amplification program was as follows: initial denaturation at 95 °C for 30 s, followed by 40 cycles of denaturation at 95 °C for 5 s and annealing/extension at 60 °C for 30 s. Melting curve analysis was performed after amplification to verify the specificity of PCR products. *CsGAPDH* (accession number: GE651107) was used as the internal reference gene. The relative expression levels of *CsCAMTA* genes were calculated using the 2^−ΔΔCt^ method. Each sample included three biological replicates and three technical replicates.

### 2.11. Statistical Analysis

All qRT-PCR data were obtained from three independent biological replicates, and each biological replicate included three technical replicates. Data are presented as the mean ± standard deviation (SD). Differences among different heat treatment time points were analyzed by one-way analysis of variance (ANOVA) using GraphPad Prism 10. Statistical significance was indicated as follows: * *p* ≤ 0.05, ** *p* ≤ 0.01, *** *p* ≤ 0.001, and **** *p* ≤ 0.0001.

## 3. Results

### 3.1. Phylogenetic Analysis of the CAMTA Gene Family in Tieguanyin

**Figure 1:**
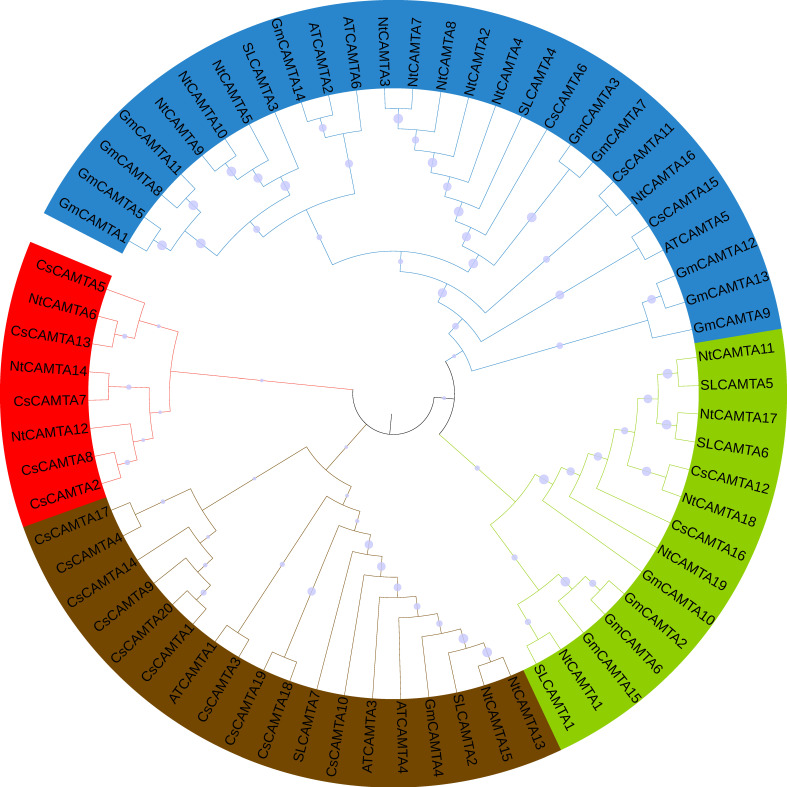
Phylogenetic analysis of *CAMTA* proteins in Tieguanyin (*Camellia sinensis* cv. Tieguanyin), soybean (*Glycine max*), tobacco (*Nicotiana tabacum*), tomato (*Solanum lycopersicum*), and *Arabidopsis thaliana*. The inner circle indicates CAMTA proteins from different species using different colors, whereas the outer colored blocks represent the four CAMTA subgroups. Branches indicate phylogenetic relationships among CAMTA proteins. Purple dots at internal nodes indicate bootstrap support values based on 1000 replicates, with larger dots representing higher support values.

This study conducted a phylogenetic analysis of *CAMTA* proteins from tea plant (*Camellia sinensis* var. *assamica*, Cs), soybean (*Glycine max*, Gm), tobacco (*Nicotiana tabacum*, Nt), tomato (*Solanum lycopersicum*, Sl), and *Arabidopsis thaliana* (At). Previous studies have reported the identification and phylogenetic analysis of *CAMTA* genes in tea plants and other plant species [26,27]. In the present study, the phylogenetic tree was constructed using the Neighbor-Joining (NJ) method with 1000 bootstrap replicates. The resulting phylogenetic tree is shown in Figure 1. In this tree, *CAMTA* proteins from different species were color-coded in the inner circle, while the outer circle distinguished the four *CAMTA* subgroups using different colors.

As shown in Figure 1, *CAMTA* proteins from the five species could be divided into four major subgroups. Several *CsCAMTA* proteins clustered closely with homologous *CAMTA* proteins from soybean, tobacco, tomato, or Arabidopsis, suggesting a certain degree of evolutionary conservation among *CAMTA* members from different plant species. Meanwhile, some *CsCAMTA* members were located in relatively independent branches, indicating possible sequence divergence within the Tieguanyin *CAMTA* gene family.

Overall, the phylogenetic analysis provides a framework for understanding the evolutionary relationships of *CsCAMTA* proteins and lays a foundation for subsequent analyses of gene structure, conserved domains, chromosomal distribution, and expression patterns under heat stress.

### 3.2. Collinearity and Physicochemical Characterization of the CsCAMTA Gene Family

**Figure 2:**
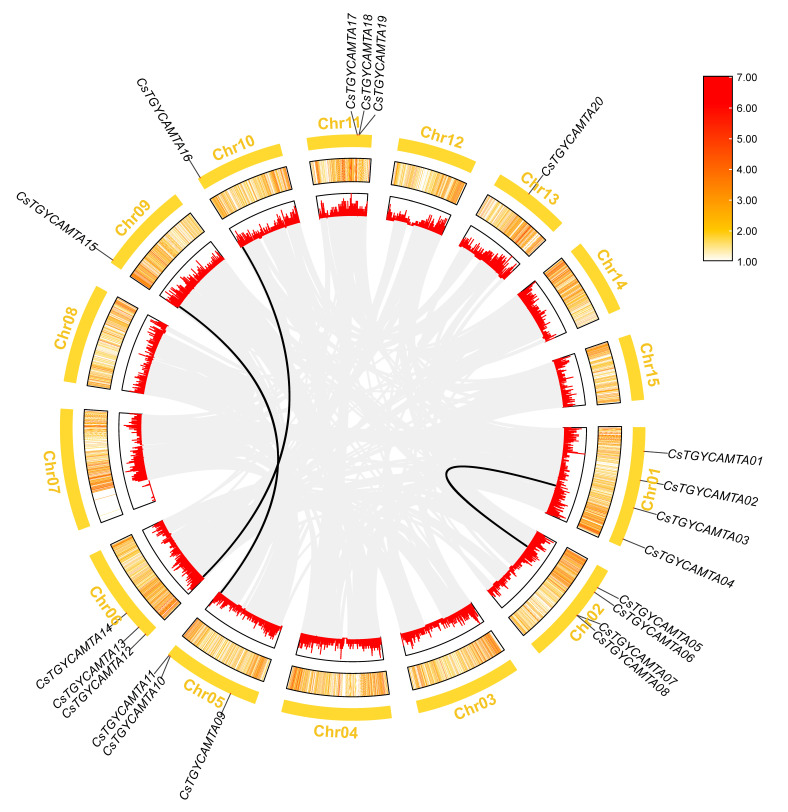
Circos diagram of *CsCAMTA* genes. The outer ideograms illustrate the tea plant chromosomes, with red peaks indicating the distribution of gene density along each chromosome. Black lines connect collinear *CsCAMTA* gene pairs, representing segmental duplication events within the Tieguanyin genome.

As shown in Figure 2, the black curves in the figure connect *CsCAMTA* genes on different chromosomes, and these links represent intraspecific collinear blocks (segmental duplication), indicating that these genes are homologous copies generated via chromosomal segmental duplication [28,29].

This collinear relationship directly supports the evolutionary conclusion that “gene duplication events drive family expansion” and clearly demonstrates the molecular mechanism underlying the expansion of the *CsCAMTA* family via segmental duplication in the Tieguanyin genome [10,28].

As summarized in Table 1, the *CsCAMTA* gene family comprises 20 members (*CsCAMTA1*–*CsCAMTA20*), which exhibit significant variations in sequence length (164–1158 amino acids) and molecular weight (17.96–128.46 kDa). This length diversity suggests differential distribution of functional domains, with longer members (e.g., *CsCAMTA3*, *CsCAMTA6*, *CsCAMTA12*) potentially possessing more complex regulatory functions. Isoelectric point analysis revealed a broad pI range (4.54–8.73), where acidic members (e.g., *CsCAMTA2*, *CsCAMTA4*) may preferentially localize to nuclear or chloroplast stroma environments, while alkaline members (e.g., *CsCAMTA9*, *CsCAMTA13*) are likely involved in DNA binding or membrane interactions. Hydrophilicity analysis indicated that the vast majority of proteins exhibit hydrophilic properties (GRAVY = −0.655 to 0.055), with *CsCAMTA17* and *CsCAMTA18* showing positive GRAVY values, suggesting the presence of potential hydrophobic domains that may participate in membrane-associated functions.

Protein stability analysis classifies family members into stable (7 members, e.g., *CsCAMTA2*, *CsCAMTA9*) and unstable (13 members, e.g., *CsCAMTA3*, *CsCAMTA7*) types, with these differences potentially arising from post-translational modifications or chaperone protein regulation. Subcellular localization predictions reveal distinct functional partitioning: nuclear-localized members (6) may primarily regulate transcription, chloroplast-localized members (7) could participate in photosynthesis and stress responses, cytoplasmic members (5) might mediate signal transduction, while the uniquely plasma membrane-localized *CsCAMTA14* may be involved in transmembrane transport. This multi-layered localization pattern fully demonstrates the functional versatility of the *CsCAMTA* family in tea plant growth, development, and environmental adaptation, providing critical insights for deeper understanding of its molecular mechanisms.

### 3.3. Structural Characterization of the CsCAMTA Gene Family in Tieguanyin

**Figure 3:**
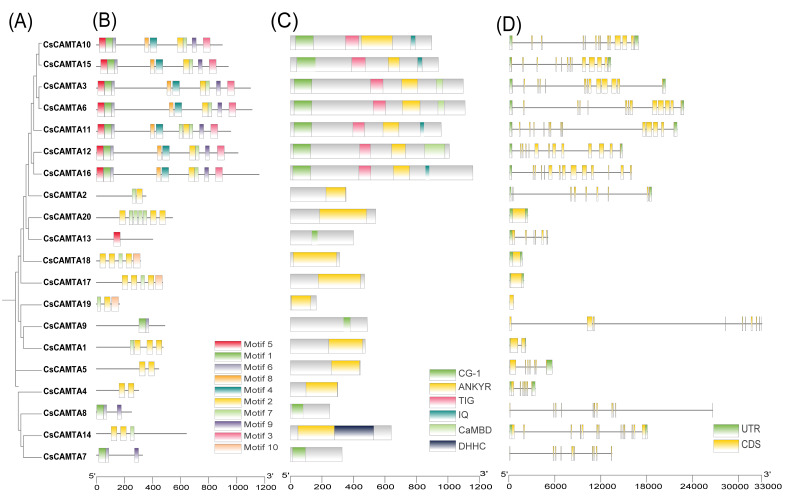
Structural analysis of the *CsCAMTA* gene family in Tieguanyin. (**A**) Phylogenetic relationships of CsCAMTA proteins. (**B**) Conserved motif distribution of CsCAMTA proteins. (**C**) Conserved domain organization of CsCAMTA proteins. (**D**) Exon–intron structures of *CsCAMTA* genes.

To investigate the structural characteristics of the *CsCAMTA* gene family in Tieguanyin tea plants, conserved motifs, conserved domains, and exon–intron structures were systematically analyzed. As shown in Figure 3, the *CsCAMTA* proteins displayed different motif compositions, conserved domain arrangements, and gene structure patterns, suggesting structural conservation and divergence among family members. Using the MEME tool, a total of 10 conserved motifs (Motif 1–10) were identified. Among them, Motif 2 was present in most family members except *CsCAMTA*7–9 and *CsCAMTA*13, suggesting that this motif may represent a relatively conserved structural feature of *CsCAMTA* proteins. Motif 3 was detected in several members, indicating possible structural conservation among specific *CsCAMTA* proteins. Motifs 5 and 8 were found in *CsCAMTA*3 and *CsCAMTA*6, whereas Motifs 7 and 9 were mainly distributed in *CsCAMTA*10 and *CsCAMTA*15, suggesting potential structural divergence among different members.

Domain analysis revealed the differential distribution of domains—ANKYR (16 members), CG-1 (11 members), TIG (7 members), and DHHC zinc finger (1 member) —and this distribution pattern reflected functional divergence. In particular, the synergistic interaction between the CG-1 domain and IQ motifs facilitated the coupling of calcium signaling with transcriptional regulation.

Gene structure analysis showed that the number of exons (1–15) varied greatly among family members, and significant variations were also observed in non-coding regions. The absence of 3′UTR in some *CsCAMTA* gene members may enhance mRNA stability, whereas the longer UTRs (>500 bp) in *CsCAMTA*17 and *CsCAMTA*20 likely contain abundant regulatory elements. Evolutionary analysis indicated that gene duplication events drove family expansion. While the conserved core motifs (Motif 1–3) and CG-1 domain maintained basic functions, the combination of specific motifs and domains, such as ANKYR and TIG domains, promoted functional divergence, enabling Tieguanyin to adapt to complex environments such as high altitude and low temperature. These findings provide important insights for further elucidating the role of *CsCAMTA* genes in environmental adaptation and quality formation in tea plants.

Overall, the integrated analysis of conserved motifs, conserved domains, and exon–intron structures indicated that the *CsCAMTA* gene family exhibits both structural conservation and divergence. These results provide useful information for subsequent expression analysis and functional characterization of *CsCAMTA* genes.

### 3.4. Chromosomal Distribution and Evolutionary Analysis of the CsCAMTA Gene Family in Tieguanyin

In this study, a total of 20 *CsCAMTA* genes were identified in Tieguanyin and unevenly distributed across eight chromosomes. The densest distributions were observed on chromosomes 1 and 2, each harboring four *CsCAMTA* members, namely *CsTGYCAMTA01*–*CsTGYCAMTA04* and *CsTGYCAMTA05*–*CsTGYCAMTA08*, respectively. Chromosomes 5, 6, and 11 each contained three *CsCAMTA* genes, whereas chromosomes 9, 10, and 13 each carried only one *CsCAMTA* gene. This uneven distribution suggests that *CsCAMTA* genes were not uniformly retained across the Tieguanyin genome.

Collinearity analysis identified three segmental duplication groups within the *CsCAMTA* gene family. As shown in Table 2, Group 1 included *CsTGYCAMTA*03, *CsTGYCAMTA*05, and *CsTGYCAMTA*06; Group 2 included *CsTGYCAMTA*10, *CsTGYCAMTA*11, and *CsTGYCAMTA*15; and Group 3 included *CsTGYCAMTA*12 and *CsTGYCAMTA*16. These duplicated gene groups suggest that segmental duplication may have contributed to the expansion of the *CsCAMTA* gene family in Tieguanyin.

Previous studies have reported that tandem and segmental duplications are important mechanisms involved in the expansion and diversification of plant gene families. Consistent with these findings, the collinearity results in this study provide useful information for understanding the evolutionary expansion of *CsCAMTA* genes in Tieguanyin. Overall, the chromosomal distribution and collinearity analyses establish a basis for subsequent analyses of *CsCAMTA* gene structure, conserved domains, and expression patterns.

### 3.5. Cross-Species Collinearity Analysis of CAMTA Genes Between Tea Plants and Other Plant Species

**Figure 4:**
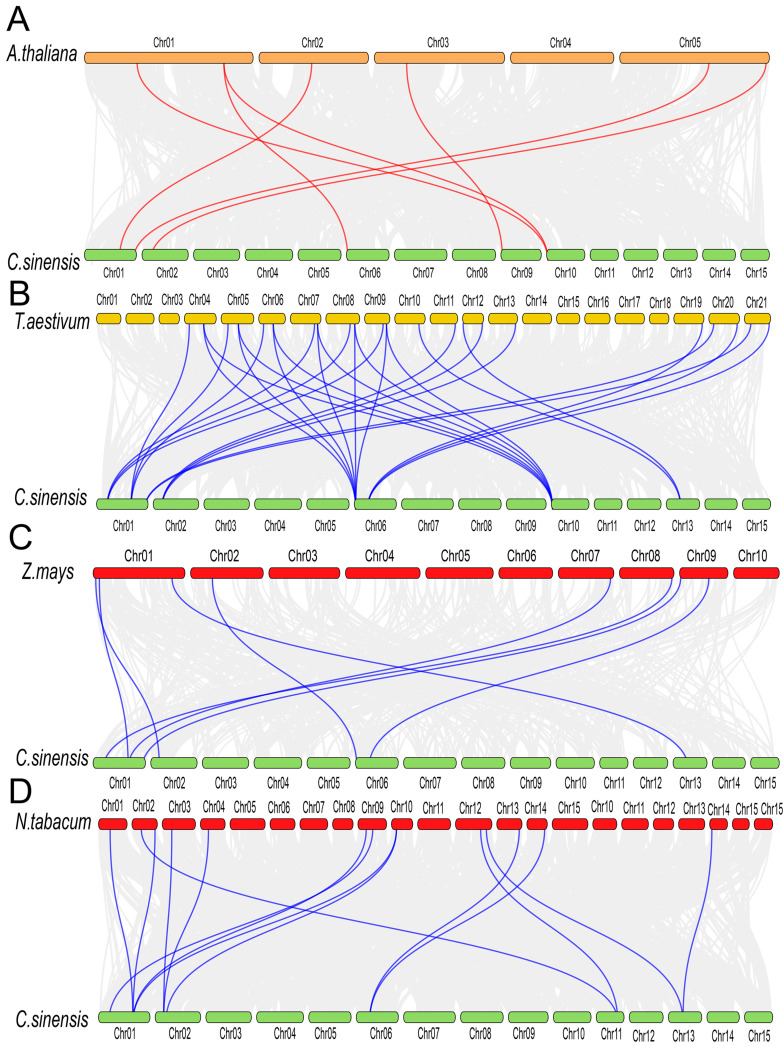
Collinearity analysis of *CAMTA* genes between *Camellia sinensis* Cv. Tieguanyin and other plant species. (**A**) Collinearity analysis of *CAMTA* genes between *Camellia sinensis* and *Arabidopsis thaliana*. (**B**) Collinearity analysis of *CAMTA* genes between *Camellia sinensis* and *Triticum aestivum*. (**C**) Collinearity analysis of *CAMTA* genes between *Camellia sinensis* and *Zea mays*. (**D**) Collinearity analysis of *CAMTA* genes between *Camellia sinensis* and *Nicotiana tabacum*. Different species names and chromosomes are represented by different colors. The red lines (in panel (**A**)) and blue lines (in panels (**B**–**D**)) represent the homologous CAMTA gene pairs between the corresponding species and the tea plant *CAMTA* genes (*CsCAMTA*s), and the gray lines represent all other homologous gene pairs on the chromosome.

In this study, multi-dimensional synteny analysis was performed to elucidate the evolutionary characteristics of the calmodulin-binding transcription activator gene family (*CsCAMTA)* in tea plants. As shown in Figure 4, interspecific synteny analysis revealed significant syntenic relationships between *CsCAMTA* genes and their homologs in species including wheat (*Triticum aestivum*), maize (*Zea mays*), Arabidopsis (*Arabidopsis thaliana*), and tobacco (*Nicotiana tabacum*) [30,31,32,33]. Specifically, syntenic genes between Arabidopsis and Tieguanyin were mainly distributed on Chr01-Chr03 and Chr05 of Arabidopsis; syntenic genes between wheat and Tieguanyin were concentrated on Chr03-Chr04 and Chr19-Chr21 of wheat; syntenic genes between maize and Tieguanyin were concentrated on Chr01-Chr02 and Chr07-Chr09 of maize; while syntenic regions between tobacco and Tieguanyin were widely distributed on Chr01-Chr04, Chr09, Chr10, and Chr12-Chr14 of tobacco. The Chr01, Chr02, and Chr06 chromosomes of Tieguanyin exhibited synteny with all four aforementioned species, suggesting that these three chromosomes are evolutionarily highly conserved core segments in the tea plant genome. The *CsCAMTA* genes carried on these chromosomes may be associated with fundamental biological processes such as calcium signal transduction and stress responses. Considering that transcription factors are widely involved in plant responses to abiotic stresses [34], these conserved chromosomal regions may provide useful candidate loci for subsequent functional validation and molecular breeding [9,28,29].

### 3.6. Analysis of Cis-Acting Elements in the Promoter Regions of CsCAMTA Genes in Tieguanyin

**Figure 5:**
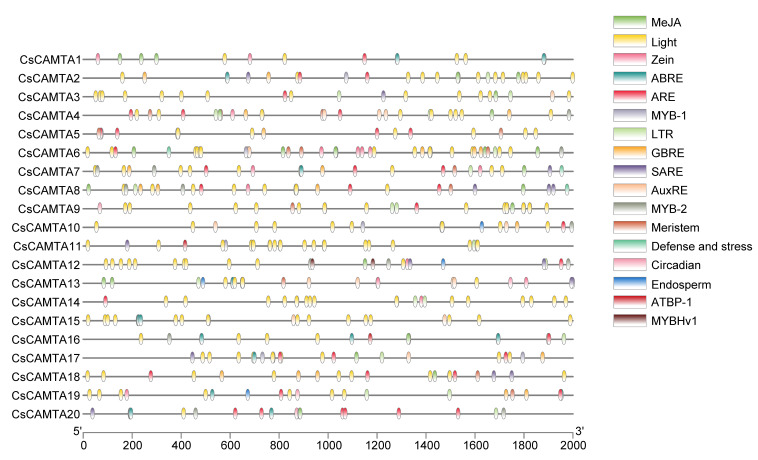
Distribution of cis-acting elements in the promoters of *CsCAMTA* genes in Tieguanyin. The legend on the right corresponds to the element types.

As shown in Figure 5, promoter analysis identified four major categories of cis-acting elements in the upstream regions of *CsCAMTA* genes, including hormone-responsive elements, stress-responsive elements, light-responsive elements, and growth/development-related elements. Hormone-responsive elements, such as ABRE, SARE, MeJA-responsive elements, GBRE, and AuxRE, suggest that *CsCAMTA* genes may be regulated by multiple hormone-related signals. Stress-related elements, including MYB-1, LTR, WUN, and ARE, were also detected in the promoter regions of several *CsCAMTA* genes, indicating their potential association with environmental stress responses. In addition, light-responsive and growth/development-related elements were widely distributed among CsCAMTA promoters. These results suggest that *CsCAMTA* genes may be transcriptionally regulated by diverse environmental and developmental signals, providing clues for further analysis of their potential roles in heat stress responses. These findings systematically revealed the multifunctional nature of the *CsCAMTA* gene family in plant environmental adaptation and growth/development, providing new directions for molecular mechanism research [35].

### 3.7. Expression Analysis of 18 CsCAMTA Genes Under Heat Stress

To further investigate the functions and expression patterns of *CsCAMTA* genes in Tieguanyin, we used Tieguanyin seedlings as experimental materials and subjected them to heat stress treatment at 40 °C. Samples were collected at 0 h, 4 h, 8 h, 12 h, and 24 h post-treatment, and quantitative real-time polymerase chain reaction (qRT-PCR) was employed to detect gene expression levels. A total of 18 representative *CsCAMTA* genes (*CsCAMTA*01–*CsCAMTA*08, *CsCAMTA*10–*CsCAMTA*19) were screened from the results.

**Figure 6:**
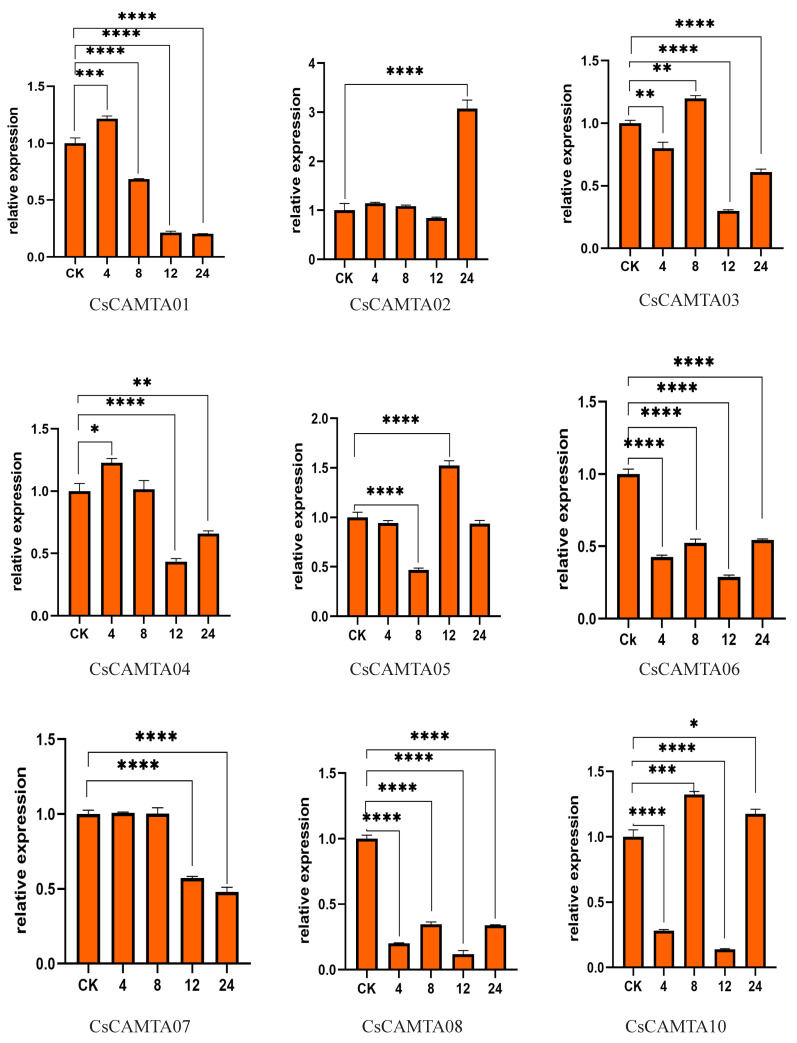

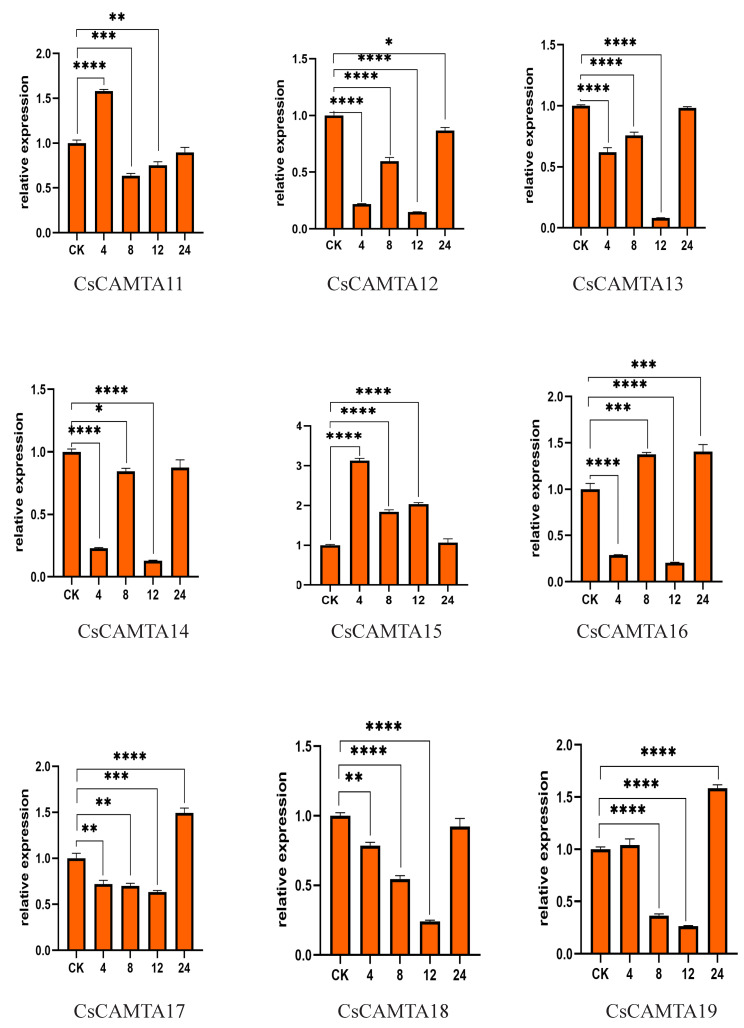
Expression patterns of 18 Tieguanyin *CsCAMTA* genes under 40 °C heat treatment. qRT-PCR was used to analyze the relative expression levels of *CsCAMTA* genes at 0, 4, 8, 12, and 24 h. Statistical significance was determined by one-way ANOVA. Asterisks indicate significant differences compared with the control: * *p* ≤ 0.05; ** *p* ≤ 0.01; *** *p* ≤ 0.001; **** *p* ≤ 0.0001.

As shown in Figure 6, the expression levels of these genes exhibited significant differences. Further analysis revealed that their expression patterns were diverse, mainly including rise first then decline, decline first then rise, and fluctuating patterns. For example, CsCAMTA1 and CsCAMTA4 were typical rise first then decline genes: their expression levels were significantly upregulated and reached peaks at 4 h post-treatment, followed by a gradual decrease. In contrast, *CsCAMTA*17 and *CsCAMTA*18 belonged to the decline first then rise type, with their expression levels peaking at 24 h post-treatment. Additionally, several special expression patterns were observed: CsCAMTA3 and *CsCAMTA*10 showed a decline-rise-decline fluctuating pattern, with expression turning points at 8 h and 12 h post-treatment; CsCAMTA7 exhibited a unique expression pattern, with no significant fluctuation in expression levels during 0–8 h post-treatment, followed by rapid downregulation during 12–24 h.

Combined with the significance labels in the bar charts of Figure 6, it can be seen that most *CsCAMTA* genes showed significant or extremely significant differences compared with the control group (CK) at least one time point after heat treatment. This indicates that these genes exhibit distinct temporal specificity in response to heat stress, and their expression dynamics are directly associated with the perception and transduction of stress signals.

### 3.8. GO Functional Enrichment and KEGG Pathway Analysis of the CsCAMTA Gene Family

**Figure 7:**
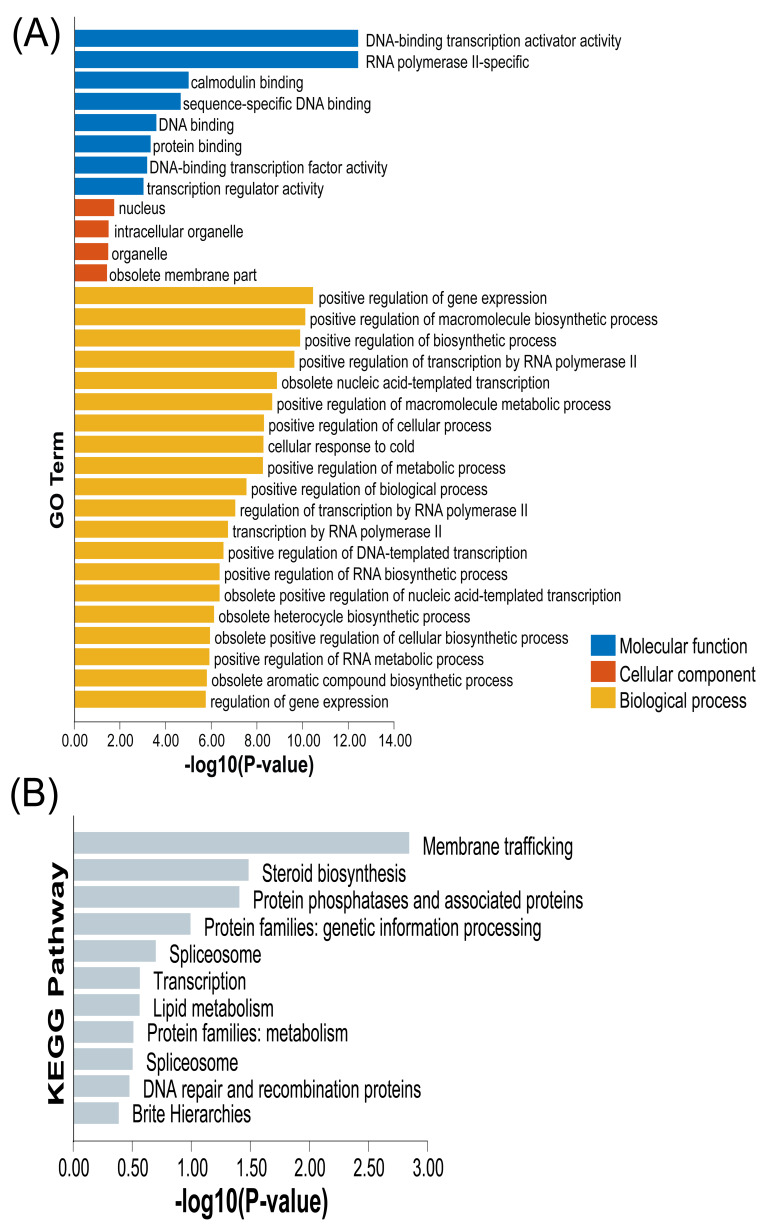
GO enrichment (**A**) and KEGG analysis of *CsCAMTA* genes (**B**).

In this study, Gene Ontology (GO) functional enrichment analysis and Kyoto Encyclopedia of Genes and Genomes (KEGG) pathway analysis were performed on *CsCAMTA* genes, and the results are shown in Figure 7. The GO classification system categorizes gene functions into three core domains: Molecular Function (blue), Cellular Component (orange), and Biological Process (yellow), with the −log_10_(*p*-value) indicating enrichment significance (higher values denote stronger enrichment). As shown in Figure 7A, in the Molecular Function category, *CsCAMTA* genes were most significantly enriched in the term *DNA-binding transcription activator activity*, *RNA polymerase II-specific* (−log_10_(*p*-value) ≈ 14), while terms including *calmodulin binding*, *sequence-specific DNA binding*, and *DNA-binding transcription factor activity* were also highly enriched. In the Cellular Component category, *CsCAMTA* gene products were primarily localized to terms such as *nucleus* and *intracellular organelle*, which aligns with the subcellular localization characteristics of transcription factors. In the Biological Process category, the most prominent term was positive regulation of gene expression, while transcription regulation-related terms such as *positive regulation of macromolecule biosynthetic process* and *positive regulation of transcription by RNA polymerase II* were also highly enriched. Additionally, *stress response terms including cellular response to cold* were significantly enriched, suggesting that this gene family is involved in plant adaptation to low-temperature stress [36].

As shown in Figure 7B, KEGG pathway enrichment analysis showed that Membrane trafficking was the most significantly enriched pathway (−log_10_(*p*-value) ≈ 3.0) [37]. In addition, pathways including Steroid biosynthesis, Protein phosphatases and associated proteins, and Protein families: genetic information processing were also relatively enriched.

## 4. Discussion

Tieguanyin is a representative oolong tea cultivar in China, and its growth, quality formation, and stress resistance are influenced by both genetic background and environmental conditions [12,38,39,40,41]. The *CAMTA* gene family encodes a group of highly conserved calmodulin-binding transcription factors that are involved in calcium signal transduction, stress responses, plant development, hormone regulation, DNA binding, and transcriptional regulation [42,43,44]. In the present study, 20 *CsCAMTA* genes were identified from the Tieguanyin genome, and their phylogenetic relationships, physicochemical properties, conserved motifs, chromosomal distribution, collinearity, promoter cis-elements, GO/KEGG annotation, and heat-responsive expression patterns were systematically analyzed.

The 20 identified *CsCAMTA* genes were unevenly distributed across eight chromosomes, suggesting that this gene family was not uniformly retained in the Tieguanyin genome. The proteins encoded by these genes varied considerably in length, ranging from 164 to 1158 amino acids, and their predicted molecular weights ranged from 17.96 to 128.57 kDa. Such variation in protein length and molecular weight may be related to differences in domain composition among family members. The theoretical isoelectric points of *CsCAMTA* proteins ranged from 4.54 to 8.73, indicating differences in predicted charge properties. Most *CsCAMTA* proteins showed negative GRAVY values, suggesting an overall hydrophilic nature, whereas *CsCAMTA17* and *CsCAMTA18* had GRAVY values close to zero. Protein instability index analysis showed that several *CsCAMTA* proteins were predicted to be stable, whereas others were predicted to be unstable [45,46]. The relatively high instability index of several *CsCAMTA* proteins suggests that these proteins may have more dynamic structural properties, which could be associated with regulatory processes such as stress response and signal transduction [47]. Subcellular localization prediction indicated that *CsCAMTA* proteins may be distributed in the cytoplasm, nucleus, chloroplast, and plasma membrane. Among them, the predicted nuclear-localized members may be associated with transcriptional regulation, which is consistent with the general characteristics of transcription factors.

To further investigate the evolutionary relationships of *CAMTA* proteins, a phylogenetic tree was constructed using *CAMTA* members from Tieguanyin, soybean, tobacco, tomato, and *Arabidopsis thaliana*. Based on the phylogenetic classification, *CAMTA* proteins from the five species were divided into four subgroups [30,31,32,33]. Several *CsCAMTA* proteins clustered closely with *CAMTA* proteins from soybean, tobacco, tomato, or *Arabidopsis*, suggesting a certain degree of evolutionary conservation among *CAMTA* members from different plant species. For example, *CsCAMTA11* and *NtCAMTA16* were located in the same clade, indicating that these proteins may share relatively close evolutionary relationships. In addition, some *CsCAMTA* members formed relatively independent branches, suggesting possible sequence divergence within the Tieguanyin *CAMTA* gene family.

Subgroup classification provides useful clues for inferring the potential functions of *CsCAMTA* genes based on homologous genes in model plants. In Subgroup I, *AtCAMTA3* has been extensively studied and has been reported to participate in plant immune responses and abiotic stress adaptation by regulating downstream target genes [12,13]. For example, *AtCAMTA3* is involved in the regulation of *EDS1*, a key gene associated with immune signaling, and also participates in pathways related to reactive oxygen species (ROS) accumulation and salicylic acid (SA) signaling [12,48,49]. Therefore, Tieguanyin *CsCAMTA* members clustered with *AtCAMTA3* in Subgroup I may have potential roles in stress-related regulatory processes. In Subgroup III, *CsCAMTA6*, *CsCAMTA11*, and *CsCAMTA15* were clustered with *AtCAMTA6*. Previous studies showed that *AtCAMTA6* is associated with salt stress responses and may participate in the regulation of sodium ion homeostasis and stress-related transcriptional networks [50]. Based on these phylogenetic relationships, *CsCAMTA* members in this subgroup may be associated with calcium- or hormone-mediated regulatory pathways.

Synteny analysis was performed to explore the evolutionary conservation of *CAMTA* genes between Tieguanyin and other plant species, including wheat, tobacco, and *Arabidopsis*. The results showed that Tieguanyin had the largest number of *CAMTA* homologous gene pairs with wheat, followed by tobacco, whereas fewer homologous gene pairs were detected between Tieguanyin and *Arabidopsis*. The relatively large number of homologous gene pairs between Tieguanyin and wheat may partly be related to the hexaploid nature of the wheat genome and the retention of homologous genes during genome evolution [51]. In addition, the syntenic relationships between Tieguanyin and tobacco suggest that some *CAMTA* genes may have been conserved during the evolution of dicotyledonous plants. Gene duplication is considered an important mechanism driving gene family expansion and functional diversification in plants [52,53]. In this study, intragenomic synteny analysis identified several segmental duplication relationships among *CsCAMTA* genes, suggesting that segmental duplication may have contributed to the expansion of the *CsCAMTA* gene family in Tieguanyin.

Gene structure and conserved domain analyses showed that most *CsCAMTA* proteins contained *ANKYR* domains [54]. The *ANKYR* domain is generally associated with protein–protein interactions and may contribute to the connection between calcium-related signaling and transcriptional regulation. In addition, members such as *CsCAMTA3*, *CsCAMTA6*, and *CsCAMTA12* contained *CaMBDs*, which are calmodulin-binding regions associated with calcium/calmodulin-mediated regulation. The presence of these domains suggests that *CsCAMTA* proteins may retain the basic structural characteristics required for calcium/calmodulin-associated transcriptional regulation [55]. Notably, *CsCAMTA14* was the only member containing a DHHC zinc finger domain, suggesting that this member may have a distinct structural feature compared with other *CsCAMTA* proteins. This domain composition provides useful information for further analysis of potential functional divergence within the *CsCAMTA* family [56,57].

Heat stress caused by global warming has become one of the major abiotic stresses limiting plant growth, development, and crop productivity [58]. Under heat stress conditions, plants regulate gene expression through complex molecular networks, in which transcription factors serve as important regulatory components [59]. In this study, qRT-PCR analysis was performed to examine the relative expression levels of 18 *CsCAMTA* genes after 40 °C heat treatment at 0 h, 4 h, 8 h, 12 h, and 24 h. The results showed that different members of the *CsCAMTA* gene family exhibited diverse temporal expression patterns, including early-induced, sustained-response, and late-induced patterns. For example, *CsCAMTA10* was upregulated at 4 h and maintained relatively high expression levels at 8 h and 12 h, whereas *CsCAMTA16* began to increase at 12 h and reached its highest expression level at 24 h. Statistical analysis showed that several *CsCAMTA* genes, such as *CsCAMTA2*, *CsCAMTA10*, and *CsCAMTA16*, exhibited significant expression changes compared with the control at one or more heat treatment time points. These expression patterns suggest that several *CsCAMTA* genes may represent candidate heat-responsive genes in Tieguanyin, which is consistent with previous findings that *CAMTA* genes in maize exhibit transcriptional responses under heat stress [60].

**Figure 8:**
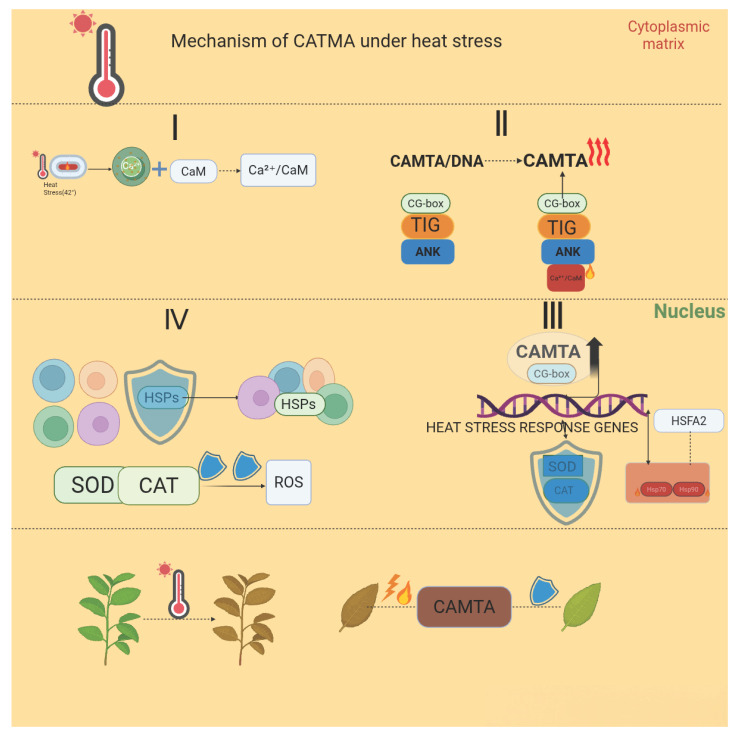
Proposed model of CsCAMTA-mediated responses to heat stress in Tieguanyin. The dashed lines divide the diagram into different cellular or functional regions, including cytoplasmic signaling, nuclear transcriptional regulation, and downstream stress-response processes. I–IV indicate the major steps of the proposed model: (I) heat stress-induced Ca^2+^/CaM signaling activation; (II) interaction between CAMTA proteins and DNA-related regulatory regions; (III) CAMTA-associated regulation of heat stress-responsive genes; and (IV) downstream responses involving antioxidant defense and heat shock protein-related pathways. Arrows indicate the putative direction of signal transduction or regulatory flow.

Plants have evolved complex molecular mechanisms to perceive and respond to heat stress, and transcription factors are important components of these regulatory networks [34,61,62]. Previous studies have shown that *CAMTA* genes participate in plant responses to various stresses, including cold, drought, salt, heat, and pathogen-related stresses. For example, *TaCAMTA* genes in wheat have been reported to respond to drought, cold, heat, and saline–alkali stresses [10,63]. Previous studies in maize showed that *ZmCAMTA* genes exhibit transcriptional responses under heat stress, supporting the potential involvement of *CAMTA* genes in plant heat stress responses [64]. These findings support the possibility that *CAMTA* genes may participate in stress responses through transcriptional regulation. Under heat stress, Ca^2+^ channels on the cell membrane can be activated, leading to transient changes in cytoplasmic Ca^2+^ concentration and the formation of calcium signals [65]. These signals are sensed by calcium sensor proteins, including calmodulin (CaM) [66]. After binding Ca^2+^, CaM undergoes conformational changes and can interact with downstream target proteins, including *CAMTA* transcription factors. Based on previous studies, *CAMTA* proteins may participate in calcium/calmodulin-mediated signaling and regulate downstream stress-related genes. In the proposed model shown in Figure 8, heat stress may activate Ca^2+^/CaM signaling, which is then connected with *CAMTA*-mediated transcriptional regulation and downstream responses such as antioxidant defense and heat shock protein-related pathways. This model provides a conceptual framework for understanding the potential involvement of *CsCAMTA* genes in heat stress responses, although the specific regulatory relationships in Tieguanyin require further functional investigation.

To explore the potential biological functions of the *CsCAMTA* gene family in Tieguanyin, GO functional enrichment and KEGG pathway analyses were performed. In the molecular function category, *CsCAMTA* genes were enriched in terms such as “DNA-binding transcription activator activity” and “calmodulin binding”, which is consistent with the typical characteristics of *CAMTA* transcription factors as calmodulin-binding transcriptional regulators [42,43,58,59]. Terms related to sequence-specific DNA binding and DNA-binding transcription factor activity were also enriched, suggesting that *CsCAMTA* genes may be associated with transcriptional regulation. In the biological process category, enriched terms included “positive regulation of gene expression” and “cellular response to cold”, indicating that *CsCAMTA* genes may be broadly involved in stress-related biological processes. Previous studies have shown that *CAMTA* genes in other plant species, such as rice, grape, and Arabidopsis, participate in stress-related regulatory pathways [19,67,68,69], which provides useful references for interpreting the potential roles of *CsCAMTA* genes in Tieguanyin.

Through KEGG pathway analysis, *CsCAMTA genes* were significantly enriched in pathways such as “Transcription”, “Membrane trafficking”, and “Steroid biosynthesis”. Among these, the enriched pathways may reflect potential links between *CsCAMTA* genes and transcriptional regulation, intracellular transport, and hormone-related processes; the coupling of Ca^2+^ signals and membrane transport is a crucial link in plant stress signal transduction. The enrichment in the “Steroid biosynthesis” pathway indicates that *CsCAMTA* may regulate the synthesis of steroid substances (brassinosteroids) in tea plants, and brassinosteroids are key hormones for stress resistance, growth, and development of tea plants [70].

## 5. Conclusions

In this study, a total of 20 *CsCAMTA* genes were identified in the Tieguanyin tea genome and systematically analyzed. These genes were unevenly distributed across eight chromosomes and showed differences in protein length, molecular weight, isoelectric point, hydropathicity, instability index, subcellular localization, conserved motifs, and domain composition. Phylogenetic and collinearity analyses suggested that *CsCAMTA* genes have experienced evolutionary conservation and possible gene duplication events during the expansion of the *CAMTA* gene family in Tieguanyin. Promoter cis-acting element analysis and GO/KEGG annotation further indicated that *CsCAMTA* genes may be associated with hormone signaling, transcriptional regulation, and stress-related regulatory processes. In addition, qRT-PCR analysis of 18 representative *CsCAMTA* genes under heat treatment revealed distinct temporal expression patterns, suggesting that several *CsCAMTA* members may act as candidate heat-responsive genes in Tieguanyin. Overall, this study provides a systematic overview of the *CsCAMTA* gene family and offers useful candidate genes for future studies on heat stress responses in Tieguanyin tea plants.

## Figures and Tables

**Table 1:** Identified *CsCAMTA* genes and their characteristics in Tieguanyin.

Gene Name	Gene ID	Size/aa	Molecular Weight/kDa	Theoretical PI	Grand Average of Hydropathicity	Instability Index	Subcellular Localization
*CsCAMTA*1	CsTGY01G0000839	474	52.14	6.1	−0.273	44.15	cytoplasm
*CsCAMTA*2	CsTGY01G0001467	352	37.94	4.54	−0.537	32.62	nucleus
*CsCAMTA*3	CsTGY01G00002159	1097	123.04	5.29	−0.655	48.66	nucleus
*CsCAMTA*4	CsTGY01G00005923	300	33.47	4.95	−0.599	40.67	chloroplast
*CsCAMTA*5	CsTGY02G00009020	443	50.11	8.19	−0.532	45.91	chloroplast
*CsCAMTA*6	CsTGY02G00011125	1108	124.53	5.9	−0.565	45.33	nucleus
*CsCAMTA*7	CsTGY02G00021169	327	37.31	6.72	−0.358	51.98	cytoplasm
*CsCAMTA*8	CsTGY02G00021172	249	27.86	7.62	−0.059	51.45	chloroplast
*CsCAMTA*9	CsTGY05G00008985	486	54.76	8.73	−0.287	35.25	chloroplast
*CsCAMTA*10	CsTGY05G00023727	896	101.38	6.66	−0.472	40.1	nucleus
*CsCAMTA*11	CsTGY05G00023086	956	106.34	7.37	−0.492	37.96	nucleus
*CsCAMTA*12	CsTGY06G00000113	1008	112.71	5.88	−0.544	48.15	nucleus
*CsCAMTA*13	CsTGY06G00006040	400	46.82	8.27	−0.568	54.58	chloroplast
*CsCAMTA*14	CsTGY06G00011553	640	69.62	6.27	−0.148	34.2	Plasma Membrane
*CsCAMTA*15	CsTGY09G00000185	939	106.05	6.77	−0.458	42.82	cytoplasm
*CsCAMTA*16	CsTGY10G00001847	1158	128.46	5.62	−0.505	47.35	nucleus
*CsCAMTA*17	CsTGY11G0001784	470	50.89	6.39	0.03	33.04	cytoplasm
*CsCAMTA*18	CsTGY11G0001847	312	33.98	6.22	0.055	25.75	chloroplast
*CsCAMTA*19	CsTGY11G0001850	164	17.96	7.79	−0.191	20.35	cytoplasm
*CsCAMTA*20	CsTGY13G00000720	541	58.32	6.54	−0.218	42.42	chloroplast

**Table 2:** Three segmental duplication groups of the *CsCAMTA* gene family in Tieguanyin.

Group	Gene ID
Group 1	CsTGYCAMTA03
	CsTGYCAMTA05
	CsTGYCAMTA06
Group 2	CsTGYCAMTA10
	CsTGYCAMTA11
	CsTGYCAMTA15
Group 3	CsTGYCAMTA12
	CsTGYCAMTA16

## Data Availability

The data presented in this study are openly available in publicly accessible repositories. The original genome sequence data of Tieguanyin tea plant (*Camellia sinensis* cv. Tieguanyin) are available in the National Center for Biotechnology Information (NCBI) under accession number JAFLEL000000000 and in the Genome Warehouse (GWH) at https://bigd.big.ac.cn/gwh/ (accessed on 3 April 2024) under the accession number GWHASIV00000000. The qRT-PCR expression data generated during this study are available from the corresponding author upon reasonable request.
